# Odontological Society of Pennsylvania

**Published:** 1898-01

**Authors:** 

**Affiliations:** Odontological Society of Pennsylvania


### ODONTOLOGICAL SOCIETY OF PENNSYLVANIA.

The regular meeting of the Society was held October 8, 1897, President Brubaker in the chair.

Upon the completion of the routine business Dr. George W. Warren read his paper on "The Therapeutic Treatment of Infected Tooth-Root Canals."

(For Dr. Warren's paper, see page 11.)

DISCUSSION.

*Dr. Litch.*—The oil of cassia has no high germicidal power, but is as nearly non-irritant as any of the series. The essential oils do not diffuse quite as rapidly as would some other antiseptic substances more nearly harmonizing with the watery constituents of the tubuli, such as sulphuric acid, or hydrochloric acid, or the peroxide of hydrogen. Nevertheless very satisfactory results can be obtained by relying solely upon the essential oils, and I often make them the basis of my antiseptic treatment.

The use of nitrate of silver is likely to produce discoloration of the teeth. The same objection is valid as regards mercurial preparations, more especially the bichloride. Nitrate of silver, however, is advantageous in certain cases of blind alveolar abscess. These abscesses, as you know, are troublesome to manage, especially those in the inferior bicuspids and molars, where we hesitate to put a drill through the bone for fear of injuring arteries or nerves. We have to drill through the apical foramen, and so draw off the contents of the sac through the canal. This is very readily accomplished, and, as a rule, gives immediate relief from the pain. After having drained the sac, however, it is difficult to treat the pulp-canal antiseptically and thereby cause healthy granulations in the tissue beyond the abscess itself. I have tried a great many remedies, especially bichloride, but have finally settled upon silver nitrate, used in such a way as to avoid or reduce to a minimum the danger of discoloration. My plan is to enlarge the opening to the root-canal with a moderate-sized drill, drain off the contents, and pass in some varnish solution, getting a film on the cavity and pulp-canal to protect it against the osmosis of the silver nitrate. Then I take a probe of considerable size and dip it in finely powdered silver nitrate, getting a portion on the end of the probe, and carry that through the pulp-canal, thus depositing it in the sac itself. The canal is then sealed temporarily with a small cone of gutta-percha. This treatment may cause some little pain, and will inevitably cause a discharge of pus and serum. But a redressing will usually cause painful symptoms to subside. After one such application we ought to trust a little to nature. In a few weeks all the discharge will probably dry up, and the canal can be filled in the usual way. I take a small cone of gutta-percha, dip it into eucalyptus oil, which is an admirable antiseptic, and thrust that up into the apex of the root. The rest of the canal is then filled with gutta-percha softened by heat. I have my instrument dipped in finely pulverized French chalk, which greatly facilitates packing.

*Dr. Truman.*—The question as to whether essential oils pass through the tubules does not interest me, for the reason that we cannot prove it. Dr. Harlan tried to demonstrate it several years ago, and he thought he succeeded. I have tried out-of-the-mouth experimentation in the laboratory, and have been unable to determine whether or not a fluid will pass through the root by simple osmotic action. You all know the experiments of Drs. Kirk, Harlan, and others in that direction, and I have found, by repeating their experiments, that it was impossible to know whether there was any fracture, cracks, or conduits through the root, and in many instances I thought that the fluid in the root would pass almost immediately through the tissue, so that I became very sceptical in that respect. But I know of no experiment in essential oils acting upon the protoplasmic fibre in those tubes that is of any special value, and I do not believe that the mere action of the oils on the decomposed portions of the pulp in the main canal is the only procedure necessary. The tubes of the teeth contain a microscopic fibre so extremely minute that while one is unimportant, yet when all the organic matter in the tubes of the tooth is taken into account it makes an amount of material that must be attended to. It is impossible to perfectly treat a tooth until the contents of these tubes are made aseptic. How is this to be done? We are not certain that essential oils will do it. I was interested when Dr. Warren said that he placed essential oils in the canal, and followed them with oxychloride of zinc. I think I demonstrated a few years ago, in regard to essential oils and coagulants, that oxychloride of zinc acting through its free chloride of zinc would coagulate the albumen in any of the tubes in which I placed it to the same extent as would pure chloride of zinc; and if that be true, and the experiments proved it, then the oxychloride of zinc and not the essential oils did the work. That is, it coagulated all filaments in the tubules throughout the body of the tooth. I know it can be done by the oxychloride of zinc, but it is equally and better done by the chloride of zinc.

In another case Dr. Warren spoke of relieving the tooth absolutely and of closing the fistula. If I understand correctly, when a tooth has a fistula it means a certain portion of the bone around the apical foramen has lost its periosteum. I hold that a tooth that has once abscessed can never be restored to health. It is no more possible to have a healthy condition result from a sequestrum in a necrosed jaw than it is from a necrosed portion of cement. In order to make a tooth perfectly healthy, treatment must be continued beyond the central canal, and there is no certainty that the essential oils will do it. But there is a certainty that the coagulants will. I demonstrated that very fully not only by capillary tubes and enlarged tubes, but I demonstrated it on teeth in the mouth, and also on teeth out of the mouth. These I examined microscopically, and invariably found that the chloride of zinc had coagulated the filaments in the tubuli to the extreme ends. Some say that coagulants will decompose under microbic influence just as well as other agents. I deny the assertion. I examined, only to-day, some albumen that had been under the effect of chloride of zinc for three years, exposed to all conditions, and decomposition was not present. It has not only changed in the test-tubes to a gelatinous mass, but it has changed in the same way in all the tubes I have treated, and if that be the case when we place a coagulating agent in a tooth and the protoplasm becomes coagulated and eventually gelatinous, the tooth always will remain clear. This is the result not only of theory, but of practice. Now, the objection to chloride of zinc has been that it will produce inflammation in the pericementum. That is true, provided it is pushed beyond the foramen. Therefore, I would not use anything but oxychloride of zinc.

Now, a word on the subject of nitrate of silver. I think I demonstrated that nitrate of silver cannot be controlled. It will penetrate into a tooth through the tubes and change the whole character of the albumen. Microscopical examination settled this to my own mind. I applied it to teeth and examined them microscopically. This also has been demonstrated by Drs. York and Bethel, although Dr. Bethel believes that nitrate of silver can be safely used where it is protected in the crown and used carefully in the root. I agree with him. It will not go beyond a certain distance in the tubes, but it is difficult to know where it will end. I would not use it at all where discoloration was an important factor.

*Dr. Peirce.*—The essayist made some allusion to the antiquity of solutions for septic canals. While I have not been in practice as long as my friend Dr. Truman, I want to say that canals were treated before we appreciated their condition. Before we had a knowledge of the micro-organisms that infected them, carbolic acid, creosote, and iodine were used. But carbolic acid, creosote, and iodine were used because they were efficient in practice. The first case I remember was a dentist bringing his wife into my office. She was suffering with an abscess. There was a fistula and a discharge of pus. The root-canals were cleaned out as thoroughly as possible, and the creosote forced through until it made its appearance at the fistulous opening. Now, Professor Truman says the parts were not restored to health. They were passive afterwards. If the tooth was previously necrosed, it could not be restored to health. Neither can we say that the space around the apical end was restored, but the abscess was entirely cured, and there was no trouble afterwards from that tooth. Now, we have all treated teeth in that way, with the result that we have brought them into a passive if not healthy condition, but they are as serviceable. I am very much in favor of Dr. Truman's chloride of zinc. I have never found any remedy so effective as this in the treatment of septic canals. But the preparation of that canal for treatment is quite as important as treatment itself. We have never had any agent presented to the profession that has given us such universal satisfaction as that of sulphuric acid in the root of a tooth for the purpose of softening tissue and enlarging the canal. It is true, chloride of zinc forced through the foramen and coming in contact with the tissue does create some irritation and give some pain. But it causes the parts to heal rapidly. At the risk of giving patients trouble for twenty-four hours I cleanse out the canal and use a strong solution of chloride of zinc, even going so far as to place in a crystal. As Dr. Truman says, in those tubuli we have dead material, and dead material that must be disposed of. But if we cleanse as nearly as possible, then sterilize with chloride of zinc, and fill with oxychloride of zinc, the septic matter from the small tubuli that runs off from that canal will be harmless. It matters but little whether the canal is filled with fibres of cotton saturated with wood creosote or fibres of cotton mixed up with the paste of oxychloride of zinc.

*Dr. Gaskill.*—My experience in treating the canals of teeth has been that the essential oils are not altogether satisfactory. I do not think that they are sufficiently penetrating or permanent. The chloride of zinc and the oxychloride of zinc have been the most satisfactory, but the prime essential in the treatment is the thorough removal of all foreign substance. After that is done it matters little what antiseptic is used, so long as it is one that is fairly efficient. Creosote seems to be more efficient than any of the essential oils. For a number of years I have filled a great many of my canals with gutta-percha, first saturating the canals with iodoform and oil of cinnamon. I have cases of six years' standing where these fillings have been successful.

*The President.*—If the Society will permit I would like to ask whether in the experience of dentists present the systemic condition of the patient at the time of treatment has any bearing upon the results that are to be expected, and if the constitutional or systemic condition requires a supporting treatment? In thinking of this condition it occurs to me that if it were in any other part of the body a physician would try to raise the health of the general nutrition as well as to treat the condition. For we know that the systemic condition will be more or less resistant to micro-organisms, and the higher the level of the system the greater will be the success of the treatment.

*Dr. Litch.*—Theoretically, it is absolutely true that systemic treatment would assist to combat all irritative influences. Practically, we are confronted with these septic conditions. They are usually urgent, requiring immediate action. It is not often practicable for us to administer general treatment in this class of cases. There is a vast difference in the susceptibility of individuals. We have some who seem to run to inflammations, and others in whom they occur with much less frequency and apparently with a great deal of difficulty, because they have thoroughly healthy constitutions.

*Dr. Truman.*—I do not think dentists usually pay any attention to general treatment. When our patients come to us the majority do not require it. But there is a class of patients that the dentists should pay more attention to,—those anæmic young ladies who range from fourteen to twenty years of age. I speak of these rather than young men, because they are more liable to weakened conditions at that period. They run to inflammations very rapidly, and one can scarcely ever treat such a case for the destruction of the pulp that pericemental suppuration does not threaten. A dentist will place arsenic in the pulp without any regard to the physical conditions. Is that right? I think not. The dentist will do all sorts of things with these teeth,—separate them with separators and produce inflammation, that has to my knowledge resulted in the worst kind of necrosis. It is certainly necessary to support the constitution, or at least to give attention to these pathological conditions that are likely to supervene when we undertake treatment of those cases.

*Dr. Peirce.*—Our attention is very frequently called to discomfort from a septic canal, when the discomfort is closely connected with some depression of the vascular systemic condition. I have had many cases where pulps have been dead and passive for months. Forty years ago I had a patient who suffered badly from small-pox. When she recovered the pulps in the central lateral incisors and bicuspids had died. There was not an external flaw in the teeth. And yet the pulps one after another had died, and had to be drilled into and removed. A very interesting circumstance followed in the cuspid. It became sore from peridontitis, and death of the pulp was apparent. I had prepared a slab and a cover. The very moment my drill went through into the chamber I placed some of the material on the slab, covered it, and the slab under the microscope was seen to be alive with micro organisms. Dr. Joseph Leidy said he would like to see the specimens immediately. I prepared a paper, and it was discussed as to how the micro-organisms, without any abrasion in the tooth, could have infected that pulp-canal. That was the first time I ever heard they were latent in the blood and only waiting for some opportunity for a pabulum to produce them in dead material. Leidy took part in the discussion. It appeared reasonable that there could be no other way for micro-organisms to infest the chambers of the teeth but through the blood, or possibly through the air when the canal was opened.

*Dr. Truman.*—Was the slab sterilized?

*Dr. Peirce.*—Yes; and the instant the material was brought out it was covered, and yet the contents were full of micro-organisms. That decomposition had taken place before the tooth was opened is certain. We had the inflammatory condition which results from putrefaction. Therefore the blood must, in a normal condition, be infected with these forms of life that are latent and go through the system all the time, and only develop offensively when opportunity offers them sufficient pabulum.

*The President.*—Will Dr. Warren close the discussion?

*Dr. Warren.*—I am pleased that the paper presented has brought about the result desired,—a free expression from others. I knew that there were several gentlemen present that had hobbies, and hoped they would be frank in presenting their views. Dr. Truman made a mistake in stating that the tooth having the fistulous opening was filled with oxychloride of zinc. It was the first one treated where this filling-material was employed. As to whether the end of a tooth is necrosed, referring to the second case, it matters little so long as nature comes to our aid in removing the trouble. We can hardly say the tooth returns to a normal condition, but, as Dr. Peirce observed, it becomes passive and useful. As I have said, the lower molar was filled with oxychloride of zinc; but whether this material played a part in the preservation of that tooth I do not know; I can testify, however, that the inflammatory condition was removed entirely by the introduction of oil of cinnamon, and that during the lady's stay in this city the gum returned to an apparently normal color. The very best testimony we can have along these lines is practical results.

Adjourned.

Joseph Head, M.D., D.D.S.,
*Editor Odontological Society of Pennsylvania.*

